# Background matching, disruptive coloration, and differential use of microhabitats in two neotropical grasshoppers with sexual dichromatism

**DOI:** 10.1002/ece3.5995

**Published:** 2020-01-11

**Authors:** Víctor Hugo Ramírez‐Delgado, Raúl Cueva del Castillo

**Affiliations:** ^1^ Posgrado en Ciencias Biológicas Unidad de Posgrado, Coordinación del Posgrado en Ciencias Biológicas UNAM Coyoacán México; ^2^ Lab. de Ecología UBIPRO Facultad de Estudios Superiores Iztacala Universidad Nacional Autónoma de México Tlalnepantla México

**Keywords:** background matching, crypsis, digital photography, disruptive coloration, grasshoppers, image analysis, sexual dichromatism

## Abstract

Cryptic coloration is an adaptative defensive mechanism against predators. Color patterns can become cryptic through background coloration‐matching and disruptive coloration. Disruptive coloration may evolve in visually heterogeneous microhabitats, whereas background matching could be favored in chromatically homogeneous microhabitats. In this work, we used digital photography to explore the potential use of disruptive coloration and background matching in males and females of two grasshopper species of the *Sphenarium* genus in different habitats. We found chromatic differences in the two grasshopper species that may be explained by local adaptation. We also found that the females and males of both species are dichromatic and seem to follow different color cryptic strategies, males are more disruptive than females, whereas females have a high background matching with less disruptive elements. The selective pressures of the predators in different microhabitats and the differences in mobility between sexes may explain the color pattern divergence between females and males. Nevertheless, more field experiments are needed in order to understand the relative importance of disruptive and background matching coloration in the evolution of sexual dichromatism in these grasshoppers.

## Graphical abstract

In this study, we found chromatic differences in two grasshopper species of the *Sphenarium* genus in different microhabitats that may be explained by local adaptation. We also found that the females and males of both species are dichromatic and seem to follow different color cryptic strategies: males depend on disruptive elements, whereas females have a high background matching with less contrasting disruptive elements. The selective pressures in these microhabitats and the differences in mobility between sexes may explain the chromatic divergence between females and males.

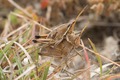

## 1 INTRODUCTION

The relationship between organisms and their environment is mediated by coloration in many ways, including social signaling, thermoregulation, protection from ultraviolet light, and antipredator defenses (Cott, 1940; Cuthill et al., 2017 and references therein). In cryptic coloration, color patterns can be adaptative if they lower the risk of being visually detected by predators. Crypsis is probably the most widespread form of concealment (Merilaita & Lind, 2005; Merilaita, Scott‐Samuel, & Cuthill, 2017). Color patterns can become cryptic by multiple mechanisms, including background coloration‐matching (colors that resemble the general color of the visual background) and disruptive coloration (patterns that conceal an animal's body outline; Merilaita, Tuomi, & Jormalainen, 1999; Norris & Lowe, 1964). Since crypsis reduces the probability of detection by predators, its variation usually matches geographic variation in substrate color (Endler, 1990; Hantak & Kuchta, 2018; Marshall, Philpot, Damas‐Moreira, & Stevens, 2015; Rosenblum, 2006; Stuart‐Fox & Ord, 2004). If females and males use different microhabitats, sexual dichromatism may evolve to better conceal them from visually oriented predators and could suggest differential crypsis values between sexes (Medina, Losos, & Mahler, 2016; Orton & McBrayer, 2019). Examples of crypsis mediating the coloration differences between females and males are found in many bird species. However, in these cases, often females are cryptic because of predation pressures, whereas males are conspicuous due to sexual selection (Badyaev & Hill, 2003; Medina et al., 2017). Nonetheless, if females and males utilize different microhabitats, natural selection for crypsis can favor the divergence between females and males in dorsal cryptic color patterns (Forsman, 1995; Forsman & Appelqvist, 1999; Medina et al., 2016).

Cryptic coloration is typical in grasshoppers (Ahnesjö & Forsman, 2006; Baños‐Villalba, Quevedo, & Edelaar, 2018; Eterovick, Figueira, & Vasconcellos‐Neto, 1997; Forsman & Appelqvist, 1999; Gillis, 1982; Karpestam, Merilaita, & Forsman, 2012), and yet no studies have addressed the evolution of sexual dimorphism in color patterns in this group of insects.

**Figure 1:**
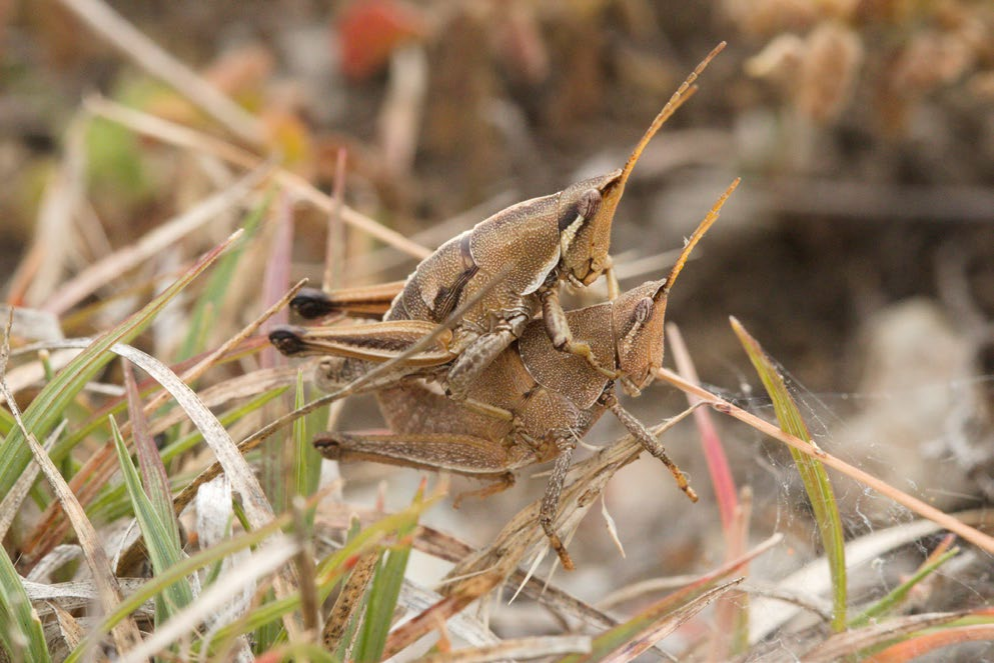
*S. purpurascens* grasshoppers. Male is mounting a female. Males have typically more bands and contrasting marking than females (photograph by Salomón Sanabria‐Urbán)

The *Sphenarium* genus is found in a wide variety of ecosystems, from northwest Guatemala to central Mexico (Sanabria‐Urbán et al., 2015). *Sphenarium purpurascens* has a broad distribution range in central Mexico and lives in a wide variety of habitats, whereas *Sphenarium planum* has flatter color patterns and only lives in the Tehuacán Valley, a xeric area with less complex background chromatic patterns (Sanabria‐Urbán, Song, Oyama, González‐rodríguez, & Castillo, 2017). They are generalist herbivores; adults are found in herbs, grass, and bush leaves. In *S. purpurascens* females are less mobile than males and can be found close to the ground, where they lay their eggs (Camacho Castillo, 1999). On the other hand, males are easier to find in higher places, looking actively for females (R. Cueva del Castillo, personal observation). They are predated by many vertebrates, including birds, mammals, and reptiles (Kevan, 1977). Distinct species within the genus have different color patterns, but in general, these grasshoppers have longitudinal and transverse bands over the thorax and abdomen, showing great continuous variation; males usually exhibit more considerable variation in patterns variation and number of bands than females (Figure 1), who tend to have color areas in more uniform tone (flatter patterns, Sanabria‐Urbán et al., 2017). Despite differences in color patterns between the sexes, there is no evidence of sexual selection acting on coloration. Males and females mate randomly with respect to male and female color patterns (Cueva del Castillo & Cano‐Santana, 2001).

In this work, we explored the potential use of disruptive coloration and background matching in males and females of two grasshopper species of the *Sphenarium* genus in different microhabitats. Because both species are found in different environments and males and females may differ in their behavior due to their different reproductive roles (Camacho Castillo, 1999; Cueva del Castillo & Cano‐Santana, 2001; Cueva del Castillo, Núñez‐Farfán, & Cano‐Santana, 1999). Due to the more complex background chromatic patterns of Pedregal de San Ángel (see below), we expected that males and females of *S. pupurascens* showed more complex color patterns than *S. planum*.

## 2 MATERIALS AND METHODS

### 2.1 Study sites and image acquisition

Images of adult grasshoppers and their backgrounds were acquired in the middle of the rainy season, during the first and second weeks of October 2017, when most of the individuals in the populations were adults and the vegetation was still green. The photographs of *S. purpurascens* were taken at Pedregal de San Ángel, Mexico City (19°19ʹ07.9ʺN, 99°11ʹ33.7ʺW), whereas the photographs of *S. planum* were taken at the Tehuacán Valley, Puebla (18°33ʹ27.9ʺN, 97°27ʹ49.1ʺW). Even though *S. pupurascens* is widely distributed in central and south Mexico (Sanabria‐Urbán et al., 2017), the Pedregal de San Ángel was chosen because its high environmental heterogeneity (see below), whereas *S. planum* was collected in the Tehuacán valley because this species has a narrow distribution (Sanabria‐Urbán et al., 2017) and lives in a more homogeneous environment than *S. purpurascens*. Both localities gave us the opportunity to test potential different cryptic strategies associated with two contrasting environments.

The Pedregal de San Ángel is a place with a complex vegetal community and complex chromatic patterns in backgrounds, situated within the Trans‐Mexican Volcanic Belt (Morrone, 2006) with a flora composition that has Neotropical and Nearctic affinities (Rzedowski, 1954; Rzedowsky, 1991). The photographs were taken in an area where the vegetation is dominated by oaks, grasses, herbs, and xerophytic scrubs. The ground is partially covered by leaf litter and black volcanic rocks. On the other hand, the Tehuacán Valley is situated in the Sierra Madre del Sur (Dávalos‐Álvarez, Nieto‐Samaniego, Alaniz‐Álvarez, Martínez‐Hernández, & Ramírez‐Arriaga, 2007), in xerophytic vegetation at the bottom of the valley (Pérez‐Valladares et al., 2019). The area where the photographs were taken is dominated by xerophytic shrubs and some small herbs (lower than 30 cm), and the ground is mainly composed of brown soil and some sedimentary rocks. In both places, an area of approximately 100 m^2^ was sampled. Three persons walked slowly over the area, searching for grasshoppers. Special care was taken to keep from disturbing any detected grasshoppers. When one was found, its location was first established, and then it was collected by hand, placed into a plastic bag (40 cm × 25 cm), and placed in a cooler until it was unable to move. Each grasshopper was returned to the same spot where it was first seen (usually on leaves or plant stems), and photographs were taken both of the dorsal view of the grasshopper and the background where it was returned. Grasshoppers that moved or escaped as a result of the approaching collectors were discarded from the study.

In all cases, photographs were taken with a Canon EOS 70D camera fitted with an 18–55 mm, f/3.5 – 5.6 lens. Camera modifications to allow sensitivity to the ultraviolet spectrum were not implemented, so our analysis is restricted to the visible spectrum. However, previous studies have shown marginal reflectance of ultraviolet light on grasshoppers (Tsurui, Honma, & Nishida, 2010). All photographs were taken under field conditions between 11:00 and 14:00 hr. in daylight. A white diffuser umbrella was placed over each grasshopper in order to remove potential shadows. All photographs were taken 40–50 cm away from the grasshopper and include a grayscale from a colorchecker card (X‐rite Colorchecker Passport Photo 2, Munsell Color Laboratories) in the same plane as the grasshoppers and their background. The grasshoppers were released at the same places where they were collected after the photographs were taken.

Following the suggestions outlined by (Stevens, Párraga, Cuthill, Partridge, & Troscianko, 2007; Troscianko & Stevens, 2015) to take objective measurements from digital photographs, we took the photographs as follows: the focal distance was constant at 55 mm, the aperture of the camera was set to f‐stops: f/5.6, the light sensitivity value (ISO) was set to 400 in all photographs, and the shutter speed was adjusted in every shot to keep from overexposing the pictures. Images were stored as . CR2 (Canon raw image format) to avoid information loss.

### 2.2 Image analyses

**Figure 2:**
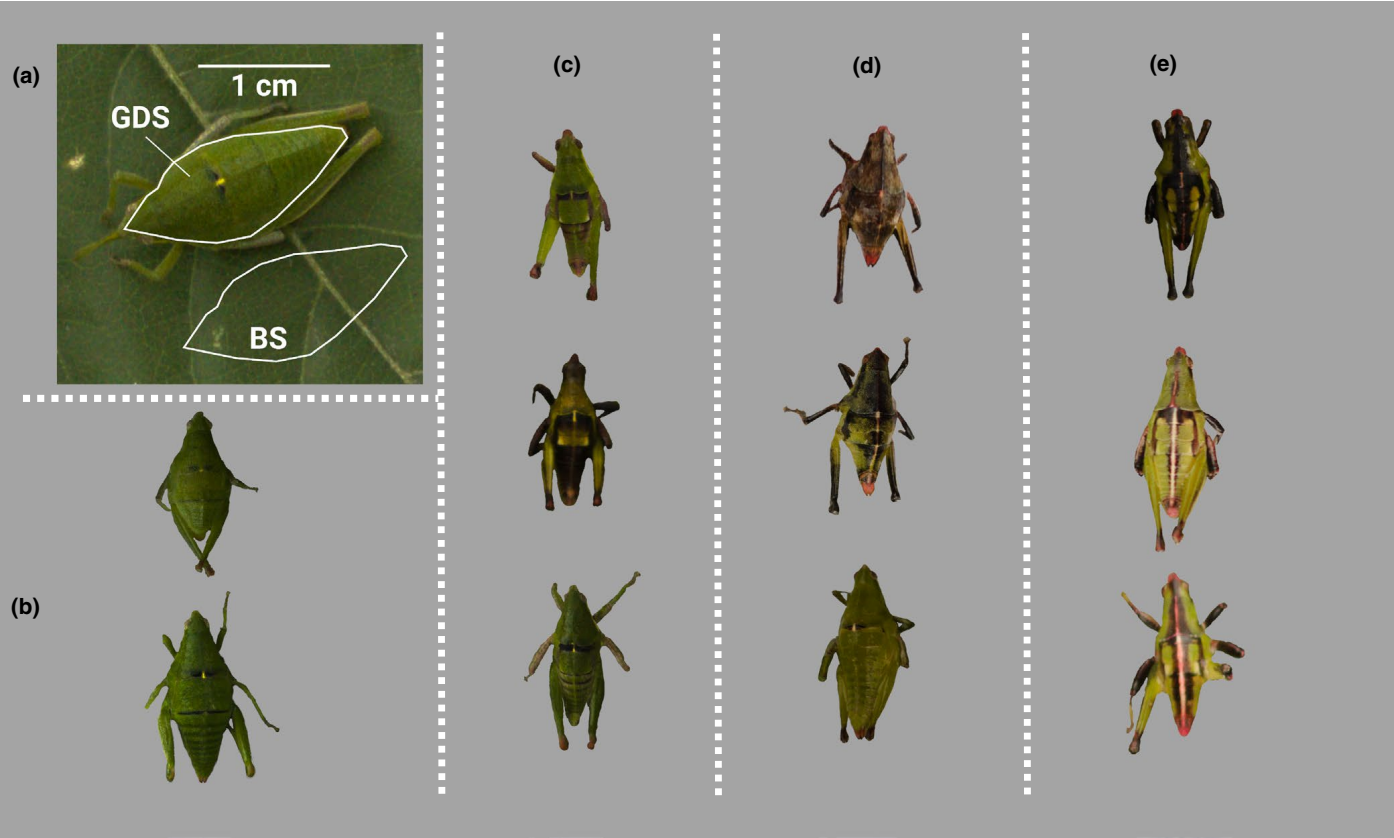
Dorsal view of *Sphenarium* grasshoppers used in this work: (a) *S. planum* female: In the image the dorsal (GDS) and background (BS) surfaces that were measured are shown. Additional images from *S. planum* (b) females, (c) males), and *S. purpurascens* (d) females, (e) males) are shown

We processed and analyzed the images with the Multispectral Image Calibration and Analysis (MICA) toolbox (Troscianko & Stevens, 2015) for ImageJ software (Schneider, Rasband, & Eliceiri, 2012). The MICA toolbox uses linear images from raw photographs and gray standard patches of the color checker as controls for different light conditions, and creates stacks of three images, known as multispectral images, corresponding to the different channels of the visible spectrum: short wave: Blue (B), mediumwave: Green (G), and longwave: Red (R) (Troscianko & Stevens, 2015). We evaluated the coloration and color patterns of each grasshopper and its background. The total dorsal surface area of the grasshoppers, excluding appendices, and a similar‐sized surface area of the adjacent background were used to obtain measurements of the grasshoppers and their microhabitats (Figure 2a). Gray reflectance standards from the ColorChecker card were applied to standardize the pictures. Photographs were scaled down to 17 pixels per mm.

#### 2.2.1 Pattern analysis

We performed a granularity analysis based on the Fast Fourier band‐pass filtering to evaluate the color patterns. Band‐pass filters allow information at different spatial scales to be separated (for details see Chiao, Chubb, Buresch, Siemann, & Hanlon, 2009; Stoddard & Stevens, 2010). Granularity analysis measures the standard deviation of pixel reflectance at different pixel scales, also known as filter sizes; this measurement is referred to as energy. The graphic representation of energy across the size of the different filters generates an energy spectrum, which is useful for comparing energy patterns between surfaces (Chiao et al., 2009). This analysis resembles how animals process the visual information, decomposing the spatial information into different spatial frequencies (Godfrey, Lythgoe, & Rumball, 1987; Stevens, 2011). Granularity analysis has been used to distinguish matches in background patterns (Chiao et al., 2009; Tyrie, Hanlon, Siemann, & Uyarra, 2015) and to mark contrasts, which are typically found in disruptive color patterns (Robledo‐Ospina, Escobar‐Sarria, Troscianko, & Rao, 2017). Granularity analysis has been used to measure the pattern markings of several species of animals, including zebras and lions (Godfrey et al., 1987), cuttlefish (Barbosa et al., 2008; Chiao et al., 2009), fish (Tyrie et al., 2015), and spiders (Robledo‐Ospina et al., 2017), as well as eggs (Stoddard & Stevens, 2010; Yang, Hu, Ma, Liang, & Møller, 2015).

We used the average pixel reflectance of red and green channels to calculate the energy spectrum of grasshoppers and their background across 15 filters ranging from 2 pixels to 256 pixels, in increments of multiples of √2. We obtained three descriptive variables from the energy spectrum: the maximum energy peak of the spectrum (*e*_max_), the filter size where *e*_max_ is reached (Filter_max_), and the proportion of the *e*_max_ compared to the rest of the spectrum (*e*_prop_), which respectively indicate contrast of the dominant marking, marking size, and pattern diversity.

#### 2.2.2 Color background matching analysis

Color background matching was evaluated by measuring individual pixel reflectance and calculating the mean reflectance values of the multispectral image for the three channels (RGB) for the grasshoppers and their backgrounds. Spectral images are in a 16‐bit scale, given this image format, the reflectance values range from zero to 65,535.

#### 2.2.3 Disruptive coloration

We evaluated the edge disruption of grasshoppers using GabRat tool implemented in MICA toolbox. GabRat tool measured the ratio between false and coherent edges of the grasshoppers' surfaces. This metric is one of the best predictors of human detection times on disruptive targets and superior to other pattern metrics algorithms tested in humans (see Troscianko, Skelhorn, & Stevens, 2018). GabRat tool is based on a Gabor band‐pass filter (see Price, Green, Troscianko, Tregenza, & Stevens, 2019; Troscianko et al., 2018). This tool estimates coherent and false edges from an object in an image. The analysis produces values ranging from zero to one. Values >0.4 are considered highly disruptive, and values <0.2 are considered low disruptive (Price et al., 2019).

For the GabRat analysis, we use the multispectral image used in the granularity analysis. We obtained the GabRat values from the photographs of grasshoppers' dorsal surface. For this analysis, the size of the Gabor filter (sigma) ideally should match the acuity of the possible viewers in order to be effective. In this study, we use a sigma value = 5 because it has been informative in analysis where the objects were scaled close to 17 pixels per mm (Price et al., 2019; Troscianko et al., 2018).

We obtained the GabRat value for R, G, B channels of the multispectral images, subsequently, we obtained the mean GabRat ($\bar{X}$ GabRat) of the three channels for every grasshoppers' photograph.

### 2.3 Statistical analyses

#### 2.3.1 Dorsal surface pattern comparisons by species and sexes

We performed a multivariate analysis of variance (MANOVA) considering species, sexes, and the interaction species × sexes to explore the *e*_max_, Filter_max_, and *e*_prop_ parameters for the dorsal surfaces of females and males of both species. Also, since the MANOVA was significant (see below), additional univariate analyses of variance (ANOVA) and honest significant differences Tukey's tests were performed to detect the significant parameters of the analysis.

#### 2.3.2 Background pattern comparisons by species and sexes

A MANOVA considering species, sexes, and the interaction species × sexes was performed to explore potential differences between the *e*_max_, Filter_max_, and *e*_prop_ parameters of the backgrounds where the grasshoppers were placed. Additional univariate ANOVAs and honest significant differences Tukey's tests were performed to detect the significant parameters of the analysis.

#### 2.3.3 Comparison between dorsal grasshopper surface and background for females and males of *S. planum* and *S. purpurascens*

For the females and males of each grasshopper species, we performed paired *t*‐tests comparing the dorsal surfaces of the grasshoppers to the background where they were placed.

#### 2.3.4 Grasshopper color background matching

In order to test the color background matching for females and males of the two grasshopper species, we performed major axis linear regressions between the color channels' reflectance of males and females of each species and their respective backgrounds. Because a high correlation and slopes near 1 between grasshoppers and their background RGB values, would denote background color matching (O'Hanlon, Feeney, Dockery, & Gormally, 2017), we compared if the slopes differed from 1 for those traits where the major axis regression was significant. We performed the slope comparations using a likelihood ratio test (details in Warton, Wright, Falster, & Westoby, 2006) in smart 3 R package (Warton, Duursma, Falster, & Taskinen, 2012).

#### 2.3.5 Disruptive coloration

An ANOVA and honest significant differences Tukey's test were performed to explore potential differences between $\bar{X}$ GabRat considering species, sexes, and the interaction species × sexes. Statistics were performed with R (R Core Team, 2018) and JMP 9.0 (2008; SAS Institute Inc.).

## 3 RESULTS

We obtained photographs of the dorsal areas and backgrounds of 35 females and 44 males of *S. planum* and 42 females and 43 males of *S. purpurascens.* All photographs were used in the color analysis. For the pattern analysis of *S. purpurascens*, 15 images were excluded because they were bellow the pixel scale requirements (Troscianko & Stevens, 2015).

### 3.1 Grasshopper dorsal surface pattern comparisons by species and sexes

**Figure 3:**
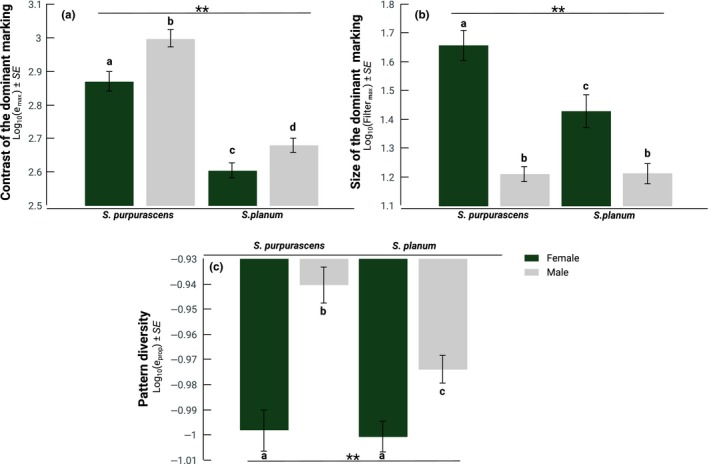
Means ± standard error of the pattern parameters for the grasshoppers’ dorsal surface: (a) *e*_max_ (contrast of the dominant marks), (b) Filter_max_ (size of the dominant marks), and (c) *e*_prop_ (pattern diversity). Bars with different letters denote differences between sexes, two stars denote differences between species according to HSD Tukey's test

The MANOVA indicates highly significant differences in the patterns' descriptive parameters (Wilks' *λ* = 0.29 *F*_3,143_ = 24.82, *p* < .0001), and the ANOVAs indicate that *e*_max_ differs between species and sexes (Table 1a). Males of both species had a higher *e*_max_ (Figure 3a), suggesting that their markings contrast more than those of females. As for the Filter_max_, we observed significant differences between species, sexes, and their interaction (Table 1b). *S. purpurascens*, and especially the females of the species, had the highest Filter_max_ values (Figure 3b), which means that the markings of *S. purpurascens*, in particular those of its females, are larger than the markings in *S. planum.* Moreover, *e*_prop_ was also highly significant (Table 1c). There were differences between both species, between females and males, and the interaction between species and sex. The males of *S. purpurascens* had the highest e_prop_ values, which suggest that the dorsal marks of *S. purpurascens* are more heterogeneous than the marks of *S. planum* males and those of the females of both species (Figure 3c).

**Table 1:** ANOVAs of (a) the *e*_max_, (b) Filter_max_, and (c) *e*_prop_ of the dorsal surface of the *Sphenarium grasshoppers*, and their backgrounds (d‐f)

Source	*df*	Sum of squares	Mean square	*F*	*p*
a. ANOVA of *e*_max_ of the dorsal surface of *Sphenarium* grasshoppers					
Species	1	3.07	3.07	134.19	<.0001
Sex	1	0.373	0.373	16.326	<.0001
Specie × Sex	1	0.025	0.025	1.103	.295
Error	145	3.318	0.022		
b. ANOVA of filter_max_ of the dorsal surface of *Sphenarium* grasshoppers					
Species	1	0.462	0.462	5.858	.017
Sex	1	3.965	3.965	50.202	<.0001
Species × Sex	1	0.475	0.475	6.01	.015
Error	145	11.454	0.079		
c. ANOVA of *e*_prop_ of the dorsal surface of *Sphenarium* grasshoppers					
Species	1	0.012	0.012	6.74	.01
Sex	1	0.065	0.065	37.167	<.0001
Species × Sex	1	0.009	0.009	5.004	.026
Error	145	0.252	0.025		
d. ANOVA of *e*_max_ of the background surface					
Species	1	1.716	1.716	30.32	<.0001
Sex	1	0.334	0.334	5.908	.016
Specie × Sex	1	0.037	0.037	0.656	.419
Error	145	8.205	0.056		
e. ANOVA of filter_max_ of the background surface					
Species	1	0.229	0.229	1.017	.316
Sex	1	0.0001	0.0001	0.0006	.982
Species × Sex	1	0.3	0.3	1.323	.252
Error	145	32.934	0.227		
f. ANOVA of *e*_prop_ of the background surface					
Species	1	0.003	0.003	0.931	.336
Sex	1	0.005	0.005	1.493	.224
Species × Sex	1	0.0002	0.0002	0.064	.801
Error	145	0.459	0.003		

Abbreviations: *df*, degrees of freedom; *F*, *F*‐values; *p*, values.

### 3.2 Background pattern comparisons by species and sexes

**Figure 4:**
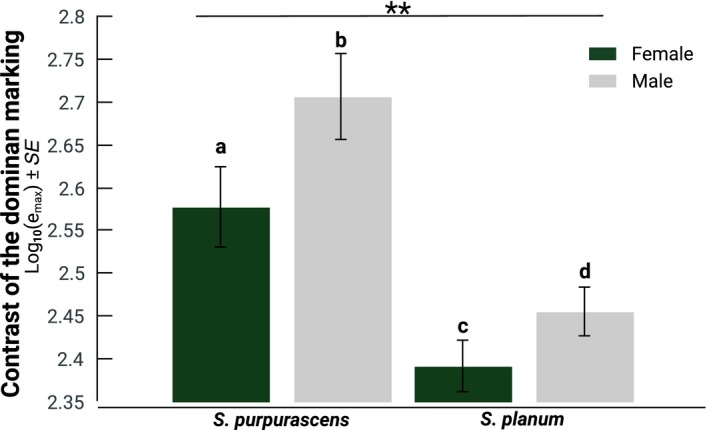
Means ± standard error of *e*_max_ (contrast of the dominant marks) for the background surface. Bars with different letters denote differences between sexes according to HSD Tukey's test, two stars denote differences between species

The MANOVA indicates significant differences in the three analyzed parameters (Wilks' *λ* = 0.78 *F*_3,143_ = 4.03, *p* < .0001). The ANOVAs indicate that *e*_max_ differs between the background associated with the species and sexes (Table 1d). The background associated with *S. purpurascens* and the males of both species had a higher *e*_max_ (Figure 4). We did not find significant differences in Filter_max_ and *e*_prop._ (Table 1e,f). These results indicate that the background in Pedregal de San Ángel has higher contrast patterns than that of the Tehuacán Valley.

### 3.3 Comparison between dorsal grasshopper surface and background for females and males of *S. planum* and *S. purpurascens*

For the females of *S. planum*, the differences between their dorsal area patterns and the background patterns where they were located were only significant for the *e*_max_ values, whereas significant differences were found in *e*_max_ and Filter_max_ for the females of *S. purpurascens* (Table 2a). For the males of *S. planum*, the differences between their dorsal area and their background were significant for the *e*_max_ and *e*_prop_ values, whereas significant differences were found in the three parameters *e*_max_, Filter_max_, and *e*_prop_ for the males of *S. purpurascens* (Table 2b). Interestingly, both females and males in Pedregal de San Ángel showed patterns that contrasted the most with their environment, which is more visually heterogeneous than the Tehuacán Valley.

**Table 2:** Paired *t*‐test comparisons of pattern parameters of the *Sphenarium* grasshoppers’ dorsal surface and their background

Species—Sex	Pattern variables	Mean (*SE*) GDS	Mean (*SE*) BS	*df*	*t*	*p*
(a) *S. planum*—♀	*e*_max_	2.605 (0.022)	2.392 (0.029)	34	5.889	<.0001
	Filter_max_	1.428 (0.057)	1.423 (0.106)	34	0.036	.971
	*e*_prop_	−1.000 (0.006)	−1.01 (0.010)	34	1.176	.247
(b) *S. planum*—♂	*e*_max_	2.680 (0.021)	2.456 (0.028)	43	7.718	<.0001
	Filter_max_	1.211 (0.036)	1.334 (0.071)	43	−1.680	.100
	*e*_prop_	0.974 (0.005)	−1.005 (0.009)	43	3.061	.003
(c) *S. purpurascens*–♀	*e*_max_	2.867 (0.028)	2.553 (0.044)	41	6.948	<.0001
	Filter_max_	1.655 (0.052)	1.4119 (0.063)	41	2.724	.009
	*e*_prop_	−0.998 (0.008)	−1.008 (0.007)	41	0.955	.345
(d) *S. purpurascens*–♂	*e*_max_	2.998 (0.025)	2.7138 (0.049)	27	6.445	<.0001
	Filter_max_	1.209 (0.025)	1.505 (0.065)	27	−4.155	.0002
	*e*_prop_	−0.940 (0.007)	−0.994 (0.011)	27	3.939	.0005

Note

Means and standard errors (*SE*) are shown.

Abbreviations: BS, background surface; *df*, degrees of freedom; GDS, Grasshoppers' dorsal surface; *p* = probability of error; *t*, *t*‐test value.

### 3.4 Grasshopper color background matching

**Figure 5:**
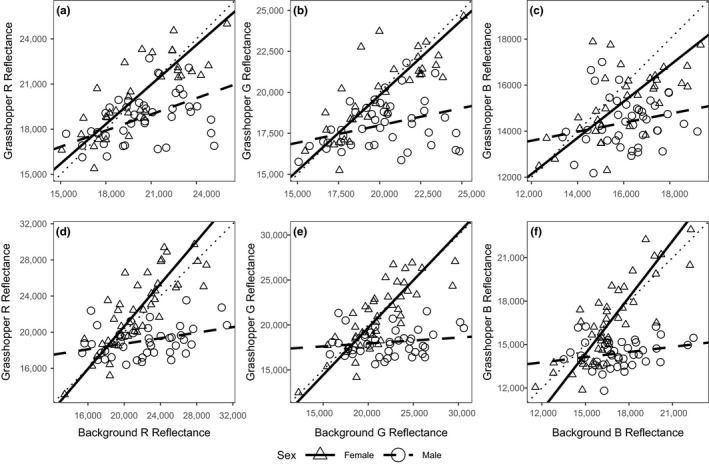
Major axis linear regression for dorsal surface RGB and background RGB reflectances for *S. planum* (a, b, c) and *S. purpurascens* (d, e, f) grasshoppers. Triangles represent females and solid lines their slopes. Circles represent males and dashed lines and their slopes. Dotted lines: *β* = 1. For details see Table 3. Scale values are in units of 16‐bit images from 0 to 65,535

In females of *S. planum* and *S. purpurascens*, the type II regressions showed a strong association between the three reflectance channels (RGB) of the dorsal area and their background. Moreover, the slopes did not differ from 1 (Table 3), which means that the females' color background matching is high. On the other hand, in the males of both species, only the R channel showed a weak association and the slope was significantly different from 1 (Table 3, Figure 5), which suggests that color background matching is much lower in males than in females.

**Table 3:** Major axis regressions for the RGB values of the grasshoppers' dorsal area (Ra, Ga, Ba) as a function of their background (Rb, Gb, Bb), for females and males of *S. planum* and *S. purpurascens*

Reflectance regressed parameters	Sex	*β*	UCL	LCL	*r* ^2^	*r*_s_	*df*	*p*
*S. planum*								
Ra to Rb	Male	0.35	0.66	0.10	0.15	−0.45	41	<.01
	Female	0.87	1.14	0.66	0.65	−0.45	32	**.32**
Ga to Gb	Male	0.21	0.48	−0.03	0.06			
	Female	0.92	1.20	0.69	0.63	−0.11	32	**.53**
Ba to Bb	Male	0.19	1.31	−0.59	0.01			
	Female	0.78	1.23	0.47	0.36	−0.18	32	**.28**
*S. purpurascens*								
Ra to Rb	Male	0.15	0.30	0.01	0.09	−0.71	42	<.0001
	Female	1.17	1.48	0.94	0.66	0.22	41	**.14**
Ga to Gb	Male	0.06	0.21	−0.08	0.01			
	Female	1.05	1.32	0.83	0.66	0.07	41	**.64**
Ba to Bb	Male	0.11	0.30	0.34	0.04			
	Female	1.29	1.62	1.04	0.68	0.01	41	<.05

Note

Slopes of the regressions (*β*), Upper (UCL; 97.5%) and Lower (LCL; 2.5%) Confidence Intervals, explained variance (*r* ^2^) are shown. In addition, *r*‐test values (*r*_s_), Degrees of freedom (*df*), and *p* values (*p*) of the analyses to test slopes different from 1 are shown for the regression that was significant. Slopes near 1 (*β *= 1) between grasshoppers and their background RGB values denote background color matching, which is indicated by no significant differences are in bold.

#### 3.4.1 Disruptive coloration

**Figure 6:**
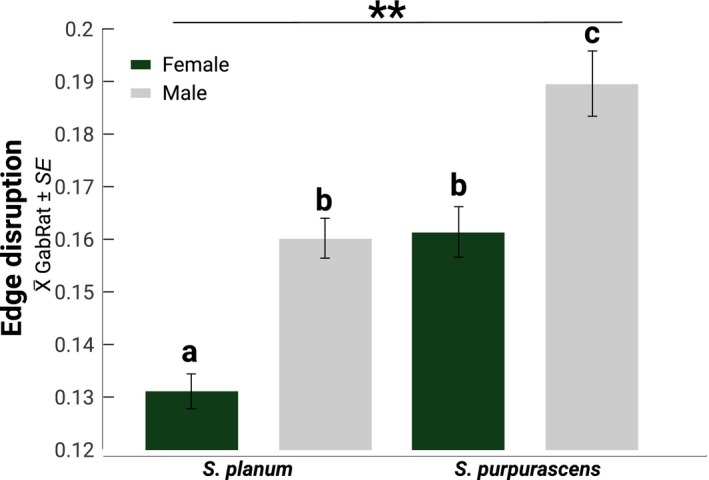
Means ± standard error of $\bar{X}$ GabRat (edge disruption) for the grasshoppers. Bars with different letters denote differences between sexes according to HSD Tukey's test. Two stars denote differences between species

The $\bar{X}$ GabRat values of the two grasshopper species are relatively low (<0.2). Nevertheless, we found significant differences between species and sexes. *S. purpurascens* is more disruptive than *S. planum* (Table 4; Figure 6), and males are more disruptive than females. Nonetheless, the interaction between both variables (species × sex) was not significant. Thus, the magnitude of the differences between males and males was similar between both species (Table 4; Figure 6).

**Table 4:** ANOVA of GabRat of the dorsal surface of the *Sphenarium* grasshoppers species

Source	*df*	Sum of squares	Mean square	*F*	*p*
Species	1	0.02604	0.02604	32.376	<.0001
Sex	1	0.03117	0.03117	38.753	<.0001
Species × Sex	1	0.00001	0.00009	0.011	.916
Error	151	0.12146	0.0008		

Abbreviations: *df*, degrees of freedom; *F*, *F*‐values; *p*, values.

## 4 DISCUSSION

Our results show differences in color patterns, chromatic differences between females and males, and matching differences between females and males and their microhabitat coloration in two *Sphenarium* grasshopper species. Moreover, we found differences in the disruptive properties between species and sexes, *S. purpurascens* is more disruptive than *S. planum*, and males across both species are more disruptive than females. The markings on males have a higher contrast, which also can indicate a disruptive function. On the other hand, females have a higher microhabitat color matching. As far as we know, this is the first study to show empirical data supporting the fact that sexual dimorphism in coloration could be associated with different cryptic strategies and microhabitat differentiation in arthropods.

The color pattern differences between *S. purpurascens* and *S. planum* can be attributed to local adaptation to different environmental conditions. *S. purpurascens* inhabits a complex environment with a wide diversity of plants, which probably leads to a wide variety of visual complexity patterns (more variety of shapes and details Dimitrova & Merilaita, 2010). This visual heterogeneity increases the possibility that both females and males were found in different background patterns. On the other hand, *S. planum* inhabits more homogeneous and less visually complex environments, which could explain why females and males were found in microhabitats with similar visual properties and had less marking patterns with less contrast.

The evolution of sexual dichromatism may be attributed to differences in the behavior of females and males associated with heterogeneous environments. *S. purpurascens* and *S. planum* seem to follow two cryptic strategies: disruptive markings and matching coloration. Disruptive coloration could evolve in visually heterogeneous microhabitats because it breaks the outlines of the organisms independently of the variable background patterns, whereas background matching could be favored in chromatically homogeneous microhabitats (Orton & McBrayer, 2019; Robledo‐Ospina et al., 2017). The marking elements associated with females and males could be cryptic if they reduce the risk of boundary detection by potential predators (Cuthill et al., 2005; Endler, 2006; Merilaita, 1998; Schaefer & Stobbe, 2006) and can be adaptative in organisms with a high mobility in heterogeneous environments (Stevens & Cuthill, 2006; Stevens, Cuthill, Windsor, & Walker, 2006). Nonetheless, this strategy is more evident in the males of both species. We could expect high mobility in males because they usually search for females actively, especially in protandrous species (Thornhill & Alcock, 1983). Interestingly, in Pedregal de San Angel, the males of *S. purpurascens* are protandrous (Cueva del Castillo & Núñez‐Farfán, 1999), and they are also more mobile than females (Camacho Castillo, 1999).

Interestingly, males of both species have the highest contrast marking in the same spatial filters (Filter_max_ 2.6) that predators may use to detect prays (Souza, Gomes and Silveira, 2011), which can reduce their risk to be detected by them. However, this hypothesis remains to be tested. Moreover, we cannot discard the idea that male coloration could be under female mate choice, even though males and females mate randomly with respect to their color patterns (Cueva del Castillo & Cano‐Santana, 2001).

In both species, the females are less disruptive than males, but their background matching is higher than males. The color‐matching with their background could lower their detectability, especially if they have reduced mobility or they are able to place themselves where the color match is high (Endler, 1978; Merilaita et al., 2017; Michalis, Scott‐Samuel, Gibson, & Cuthill, 2017). In the Tehuacán Valley, the payoff for this strategy by females could be higher due to the environmental homogeneity. However, in a heterogeneous environment, it would depend on the individuals' ability to stay in a high matching microhabitat and/or reduce their mobility (Bond, 2007; Merilaita et al., 1999), as in fact occurs in the population of *S. purpurascens* in Pedregal de San Angel (Camacho Castillo, 1999). The sexual size dimorphism bias to females (Cueva del Castillo et al., 1999) and an increase in weight due to egg maturation can explain the lack of mobility of females. Moreover, environmental heterogeneity could explain the diversity of the females' colors in Pedregal de San Angel. However, it must be tested whether females can evaluate and resettle themselves where their color match is high.

We must point out that our results are interpreted from a human visible spectrum perspective. Spectral sensitivity can be very different in other possible predators such as birds or mice, and their prey detectability could involve elements that we did not consider in this study (Théry & Gomez, 2010). However, part of the human visible spectrum overlaps with the possible visible spectrum from other predators. Thus, mice use part of the visual human spectrum (green and red) to detect potential prays (Denman et al., 2018), and under certain conditions, birds and humans have shown similar performance in detection tasks (Dukas & Kamil, 2001; Michalis et al., 2017). Further studies on predation and escape behavior are needed to test the effectiveness of both coloration cryptic strategies that we suggest in this report.

## CONFLICT OF INTEREST

None declared.

## AUTHOR CONTRIBUTIONS

Both authors contributed equally to the conception and study's design, fieldwork, data analyzes, and writing of the paper.

## Data Availability

Supplementary data associated with this study can be found at: https://datadryad.org/stash/dataset/doi:10.5061/dryad.vhhmgqnpx.
